# Towards Biohybrid Lung: Induced Pluripotent Stem Cell Derived Endothelial Cells as Clinically Relevant Cell Source for Biologization

**DOI:** 10.3390/mi12080981

**Published:** 2021-08-19

**Authors:** Michael Pflaum, Julia Dahlmann, Lena Engels, Hossein Naghilouy-Hidaji, Denise Adam, Janina Zöllner, Annette Otto, Sabrina Schmeckebier, Ulrich Martin, Axel Haverich, Ruth Olmer, Bettina Wiegmann

**Affiliations:** 1 Department of Cardiothoracic, Transplantation, and Vascular Surgery, Hannover Medical School, Carl-Neuberg-Str. 1, 30625 Hannover, Germany; Pflaum.Michael@mh-hannover.de (M.P.); Dahlmann.Julia@mh-hannover.de (J.D.); an.engels@uke.de (L.E.); NaghilouyHidaji.Hossein@mh-hannover.de (H.N.-H.); adam.denise@mh-hannover.de (D.A.); Zoellner.Janina@mh-hannover.de (J.Z.); Otto.Annette@mh-hannover.de (A.O.); sabrina.schmeckebier@gmx.de (S.S.); Martin.Ulrich@mh-hannover.de (U.M.); Haverich.Axel@mh-hannover.de (A.H.); Olmer.Ruth@mh-hannover.de (R.O.); 2 Lower Saxony Centre for Biomedical Engineering, Implant Research and Development (NIFE), Stadtfelddamm 34, 30625 Hannover, Germany; 3 Leibniz Research Laboratories for Biotechnology and Artificial Organs (LEBAO), Hannover Medical School, Carl-Neuberg-Str. 1, 30625 Hannover, Germany; 4 German Center for Lung Research (DZL), BREATH, Carl-Neuberg-Str. 1, 30625 Hannover, Germany

**Keywords:** biohybrid lung, induced pluripotent stem cells, endothelialization, hemocompatibility, EC activation, membrane oxygenator, hollow fiber membrane

## Abstract

In order to provide an alternative treatment option to lung transplantation for patients with end-stage lung disease, we aim for the development of an implantable biohybrid lung (BHL), based on hollow fiber membrane (HFM) technology used in extracorporeal membrane oxygenators. Complete hemocompatibility of all blood contacting surfaces is crucial for long-lasting BHL durability and can be achieved by their endothelialization. Autologous endothelial cells (ECs) would be the ideal cell source, but their limited proliferation potential excludes them for this purpose. As induced pluripotent stem cell-derived ECs enable the generation of a large number of ECs, we assessed and compared their capacity to form a viable and confluent monolayer on HFM, while indicating physiologic EC-specific anti-thrombogenic and anti-inflammatory properties. ECs were generated from three different human iPSC lines, and seeded onto fibronectin-coated poly-4-methyl-1-pentene (PMP) HFM. Following phenotypical characterization, ECs were analyzed for their thrombogenic and inflammatory behavior with or without TNFα induction, using FACS and qRT-PCR. Complementary, leukocyte- and platelet adhesion assays were carried out. The capacity of the iPSC-ECs to reendothelialize cell-free monolayer areas was assessed in a scratch assay. ECs sourced from umbilical cord blood (hCBECs) were used as control. iPSC-derived ECs formed confluent monolayers on the HFM and showed the typical EC-phenotype by expression of VE-cadherin and collagen-IV. A low protein and gene expression level of E-selectin and tissue factor was detected for all iPSC-ECs and the hCBECs, while a strong upregulation of these markers was noted upon stimulation with TNFα. This was in line with the physiological and strong induction of leukocyte adhesion detected after treatment with TNFα, iPSC-EC and hCBEC monolayers were capable of reducing thrombocyte adhesion and repopulating scratched areas. iPSCs offer the possibility to provide patient-specific ECs in abundant numbers needed to cover all blood contacting surfaces of the BHL with a viable, non-thrombogenic and non-inflammatory monolayer. iPSC-EC clones can differ in terms of their reendothelialization rate, and pro-inflammatory response. However, a less profound inflammatory response may even be advantageous for BHL application. With the proven ability of the seeded iPSC-ECs to reduce thrombocyte adhesion, we expect that thrombotic events that could lead to BHL occlusion can be avoided, and thus, justifies further studies on enabling BHL long-term application.

## 1. Introduction

According to the World Health Organization, end-stage lung diseases (ELD) are one of the leading causes of death worldwide with an increasing prevalence and incidence. More than 210 million patients already suffer from chronic obstructive pulmonary disease, of which about 3 million patients die per year [1], while reliable and long-lasting therapy options for ELD are limited. Lung transplantation (LTx) as the only current curative therapy option can only be offered to a few patients due to the increasing mismatch between potential organ donors to organ recipients. Moreover, difficult surgical procedures, ischemia-reperfusion injury and the induction of acute and chronic rejection result in the sobering 5-year survival rate of 56% after LTx [2]. So far there has been a lack of veritable and durable alternative therapy options to LTx which requires the development of new strategies in order to develop such solutions as they have already been implemented with the left ventricular assist device for terminal heart failure or renal dialysis in terminal renal failure. 

Therefore, we aim for the development of the implantable biohybrid lung (BHL) as alternative to LTx but also as final destination therapy, based on the technology of extracorporeal membrane oxygenation (ECMO). ECMO is currently applied within respiratory failure, as it can not only support, but also take over the whole gas exchange function of the lungs [3,4,5]. In order to achieve this, ECMO is made up of a multitude of gas exchange hollow fiber membranes (HFM), in which an oxygen-rich gas atmosphere is supplied while on the outside the blood passes by for the gas exchange via the partial pressure gradient. Nevertheless, serious limitations of this valuable technology prevent its use for long-term support. While complications due to handling and monitoring of the complex extracorporeal circuit components during ECMO are reported (e.g., dislocation of the cannula [6] or limb ischemia [7]), most frequent limitations originate from the severe interactions arising from the inevitable contact of the circulating blood and the artificial surfaces. Despite [8] strict systemic anticoagulative therapy, coagulation cascade can still be activated, resulting in increased thrombus formation [9], and the activation of the complement system [10,11] with subsequent release of inflammatory mediators and progressive adsorption of blood plasma proteins [12], aggravating the coagulation. In the course of these processes, the blood flow and thus the gas exchange via the HFM is impeded, which necessitates a life-threatening exchange of the ECMO system. In order to mitigate contact-activated thrombus formation, surface coatings of the blood contacting circuit components and the gas exchange membrane with passive (e.g., phosphoryl choline or albumin) or active molecules (e.g., heparin), or a combination of both, have been introduced by manufacturers of oxygenators in recent years. However, those coatings certified for clinical application today are only efficient to delay the undesired blood-surface interaction. Novel approaches such as coating with superhydrophobic [13,14], omniphobic [15] and further molecules are still the subject of current research and development [16,17] and have not entered the clinical arena yet. 

Towards BHL development, we aim to biofunctionalize all blood contacting surfaces to improve hemocompatibility for long-term application by the establishment of a confluent endothelial cell (EC) monolayer [18]. Thereby, the monolayer should shield the foreign material from adhesive blood components, while proteins and factors synthesized and exposed by the ECs, should contribute to suppress thrombus formation. In recent years, the general feasibility of this biofunctionalization approach has been demonstrated by our group and further groups worldwide [19,20,21,22,23,24,25,26,27,28]. In particular, stable EC adhesion and monolayer formation on hydrophobic HFM could be achieved by precoating the surface with molecules mediating cell attachment and anticoagulative properties, like heparin/albumin [19,20,21], cRGD-peptides [24], titanium dioxide [23] and fibronectin [25]. Moreover, it could be proven that the gas exchange properties of a membrane were not affected by the EC-monolayer [26,29]. Among these coatings, fibronectin in particular offers some advantages as the coating procedure does not involve multiple processing steps and can be applied using an in-house protocol to achieve homogenous HFM coating, necessary for confluent endothelialization. Additionally, fibronectin is a constituent of the vascular basement membrane, providing close-physiological adhesion sites for the ECs.

With respect to future clinical application of the BHL, primary autologous ECs would be the ideal cell source with regard to immunological compatibility. However, as all blood contacting surface areas, including the oxygenator with the HFM, the housing and tubing, result in a surface area of more than 2.5 m^2^ to be endothelialized, the low proliferation capacity and recovery rate make it impossible to generate sufficient numbers of these ECs. Therefore, studies were carried out using ECs from xenogeneic [18,19] or rather allogeneic primary cell sources [19,20,21,27,28,29], though initiating severe immune response resulting in the rejection of the ECs. In order to avoid the need for immunosuppressive therapy and cellular rejection, allogeneic HLA-silenced ECs were identified as suitable cell source for HFM endothelialization [30]. However, in general it was reported that primary ECs isolated from adult tissues tend to augment chromosomal aberrations during prolonged in vitro propagation, which might result in the loss of specific cell functions and increase the risk for tumorgenesis [31,32,33]. 

Human induced pluripotent stem cells (iPSCs) with their unlimited in vitro expansion and differentiation potential represent a valuable tool for disease models [34], drug discovery [35] as well as cell therapy approaches [36]. As iPSCs can be generated from patient samples obtained with minimal or non-invasive interventions, like peripheral blood [37], urine [38] or skin biopsies [35], expanded and differentiated towards ECs under defined [39] and scalable conditions [40] in theoretically unlimited numbers, these cells could represent an alternative cell source for BHL development, as they can also be autologous or immunocompatible by utilizing HLA-edited iPSC lines [41]. Modern methods of genetic engineering would also make it possible to equip these cells with other desirable functionalities [42,43] which can foster their capacity to prevent thrombus formation and subsequent occlusion of the BHL, e.g., by overexpression of anti-thrombogenic genes, such as nitric oxide synthase 3 (NOS3) [44,45] or thrombomodulin [46,47]. Hence, we analyzed the general suitability of iPSC derived EC (iPSC-ECs) for HFM endothelialization, in particular their capacity to form a viable and confluent monolayer on HFM, indicating physiologic endothelial cell specific anti-thrombogenic and anti-inflammatory properties to ensure future long-lasting BHL application with reduced or even omitted systemic anticoagulative therapy.

## 2. Materials and Methods

### 2.1. General Remarks 

All cell cultures were conducted at 37 °C in a cell culture incubator at 5% CO_2_ and saturated humidity. Human tissue was obtained after approval by the local Ethics Committee (Ethical approval Nos.: 844-2010 and 9271_BO_K_2020) and following the donor’s written informed consent, or in the case of newborns, following informed consent of the parents. Primary control ECs were obtained from human umbilical cord blood (hCBECs) and cultivated as described previously [19].

### 2.2. Generation and Cultivation of Human iPSCs

Human iPSCs were generated from three different sources and donors. Donor1-iPSCs were derived from human cord blood by lentiviral reprogramming [37], Donor2-iPSCs were derived from human cord blood CD34(+) cells with Sendai virus as vehicles for reprogramming factor delivery, MHHi001-A [39] and Donor3-iPSCs were derived from human peripheral blood, likewise with the use of Sendai viruses, MHHi008-A [48]. Prior to differentiation, iPSCs were maintained as feeder-free monolayer cultures on Geltrex-coated flasks in E8 medium. Cells were passaged every 3–4 days using StemPro Accutase (Life Technologies GmbH, Darmstadt, Germany) and seeded at 5 × 10^4^ cells/cm^2^ in E8 medium including 10 µM Y-27632 [49] (LU Hannover) for the first 24 h after seeding.

### 2.3. Generation and Purification of iPSC-Derived ECs

iPSC-differentiation to ECs was conducted in scalable suspension cultures in 125 mL Erlenmeyer shaker flasks (Corning, Kaiserslautern, Germany) on an orbital shaker (Celltron, Infors GmbH, Einsbach, Germany) placed in a cell culture incubator at 70 rounds per minute as described previously [40]. In brief, undifferentiated iPSCs were inoculated at 5 × 10^6^ cells/20 mL in E8 medium including 10 µM Y-27632 and precultured for 24 h to achieve aggregate formation. Differentiation was started with mesoderm induction in N2B27 medium including 25 ng/mL BMP4 and 7.5 µM CHIR99021. After 72 h of mesoderm induction the medium was switched to Stempro34 medium freshly supplemented with 2 µM forskolin and 200 ng/mL VEGF to induce endothelial differentiation. On day 7, aggregates were dissociated with collagenase II (Worthington, PAN Biotech, Aidenbach, Germany). To remove remaining cell clumps, the suspension was filtered using a 100 µm cell strainer. CD31 positive cells were purified using the CD31 MicroBead Kit (Miltenyi Biotech, Bergisch Gladbach, Germany) according to the manufacturer’s instructions. A purity of >95% CD31 positive cells was considered sufficient to carry out further experiments.

### 2.4. Cultivation of ECs on Tissue Culture Plastic and PMP Membranes

For expansion of primary and iPSC-derived ECs, cells were cultivated on fibronectin-coated (FN; 2.6 µg/cm^2^, Corning, Kaiserslautern, Germany) tissue culture plastic (TCP; Delta-surface, Nunc, Waltham, MA USA) in EGM-2 medium (2% fetal calf serum, LONZA, Cologne, Germany) supplemented with 1% (*v*/*v*) penicillin/streptomycin (Thermo Scientific, Waltham, MA USA) as detailed in Olmer et al. [40]. iPSC-derived ECs were expanded on TCP over 3–4 passages before seeding on fibronectin-coated PMP membranes. Therefore, iPSC-ECs were split every 3–4 days using Accutase (Thermo Scientific, Waltham, MA USA) and seeded at a 1:3 ratio. For cultivation on ECMO gas exchange surface material, PMP membranes were cut from 50 µm thick PMP membrane foil (Goodfellow, Hamburg, Germany) into round discs and mounted into 6-well plates by applying silicon forms (rema-sil, Dentaurum, Ispringen, Germany) molded to fit the wells leaving a surface area of 3.14 cm^2^ and coated with FN. Cells were seeded on PMP membranes at constant densities of 1.25 × 10^4^ cells/cm^2^ and cultivated in EGM-2 for 3–4 days for TNFα assays. 

### 2.5. Cultivation of ECs on PMP HFM

The seeding of PMP HFM with ECs was carried out using a modified protocol described previously [21]. Rectangular samples (3.2 × 1.8 cm^2^, 40 parallel aligned hollow fibers) were cut from HFM meshes (Oxyplus, Membrana, Wuppertal, Germany), immersed in a fibronectin solution (25 µg/mL) over night at 4°C and mounted in custom made frames made of polycarbonate. For seeding with ECs, assembled HFM in frames were placed in 50 mL perfusion syringes (B. Braun, Germany) filled with 25 mL EC/EGM-2 suspension (1.3 × 10^5^ ECs/mL). Cell adhesion to the HFM surface was allowed for 4 h at 37 °C, while syringes were rotating horizontally (1 rpm). After seeding, framed HFM samples were placed in culture dishes and incubated EGM-2 under static culture conditions for 48 h. A schematic illustration depicting the seeding setup can be found in Supplementary Figure S1. 

### 2.6. Cryo-Sectioning and Immunofluorescence Staining of Seeded HFM

For the immune-fluorescence detection of cellular components surrounding the hollow fibers, pieces of the endothelialized HFM mesh were excised using a skin biopsy punch cutter (0.8 cm in diameter), fixed in methanol or 4% PFA for 10 min and either placed as patches into the wells of glass bottom dishes (Mattek, Ashland, MA USA) containing PBS or embedded in TissueTek-O.C.T. compound (VWR, Hannover, Germany) on dry ice for cryo-sectioning. Sections with a thickness of 14 µm were generated by using a cryo-microtome (Mikrom HM 560, Thermo Fisher Scientific, Germany). Double-sided, transparent glue tape (300LSE, 70 µm thick, 3M, Neuss, Germany) was applied on the glass slide to prevent the detachment of the sections during the staining procedure. 

For the detection of the intercellular adherence junction protein VE-cadherin, sections and patches of PFA-fixed HFM were used. Sections and patches from methanol fixed samples were forwarded to collagen type-IV staining. After washing with Dulbecco’s phosphate-buffered saline (DPBS), ECs were permeabilized and blocked against unspecific antibody binding by incubation with 0.25% Triton-X100, diluted in Tris-buffered saline supplemented with 5% serum (of the respective host of the secondary antibody) for 20 min at RT. The sections and patches were then rinsed three times with PBS and incubated with primary antibodies, in 1% bovine serum albumin (BSA) in PBS w/o Ca^2+^/Mg^2+^; for 1 h, at RT. After three washing steps, fluorescence-labeled secondary antibodies were applied for 1 h, at RT in the dark. A list of used antibodies can be found in Table S1. Nuclei were stained with the Höchst33342 dye (10 µg/mL) added to the secondary antibody incubation for the last 30 min. Sections incubated with isotype-matching antibodies were prepared as controls in parallel. After embedding in mounting medium (Immumount, Dako, Hamburg, Germany), microscopy images were obtained using a fluorescence microscope (AxioVert A1, Zeiss, Jena, Germany) and a camera (AxioCam ICm1, Zeiss, Germany) or a confocal laser scanning microscope (CLSM SP8-system, Leica Microsystems, Jena, Germany). Images acquired with the CLSM are depicted as maximum projection from at least 10 focus planes per region. 

### 2.7. TNFα Assay

Confluent EC layers on PMP membranes were incubated with 10 ng/mL tumor necrosis factor alpha (TNFα, Bachem, Bubendorf, Switzerland) diluted in EGM-2 medium for 6 h. Untreated cells served as controls. After stimulation, cells were washed once with PBS w/o Ca^2+^ and Mg^2+^ and used for flow cytometry or qPCR analysis.

### 2.8. Flow Cytometry

ECs were detached from PMP membranes with Accutase (Thermo Scientific). Cells were incubated for 30 min at 4 °C with directly labelled antibodies or isotype controls (Table S1) diluted in flow buffer (PBS + 0.5% BSA and 2 mM EDTA). Antibodies used for flow cytometry can be found in the online supplementary data. Flow cytometry analysis was performed using a MACSQuant Analyzer10. Quantification of mean fluorescence intensities (MFI, represents geometric mean of samples normalized to corresponding isotype controls) was performed using FlowJo v10.5.3.

### 2.9. RNA Isolation and Quantitative Real-Time Polymerase Chain Reaction (qRT-PCR)

Total RNA was prepared from ECs grown on PMP membranes after stimulation with TNFα and used for cDNA synthesis. qRT-PCR was performed to assess expression levels of E-selectin, ICAM-1, VCAM-1, tissue factor and thrombomodulin, all relative to the reference gene β-actin. Detailed specifications on the experimental procedures and the primers (Table S2) used can be found in the online supplementary data.

### 2.10. Leukocyte Adhesion Assay

Human promyelocytic leukemia cells (HL-60) were raised in suspension culture in RPMI 1640 (VLE-RPMI 1640, Biochrom AG, Berlin, Germany) supplemented with 10% (*v*/*v*) FBS. The cell density was kept between 0.1 × 10^6^ and 0.9 × 10^6^ cells/mL. HL-60 cells were labelled with 25 µM cell tracker red dye (Thermo-Fisher, Germany), following the manufacturer’s instructions. Confluent endothelialized PMP membranes with and without deliberate TNFα activation were supplemented with 10^6^ labelled HL-60 cells, added to the EC culture medium and incubated on a rocking platform for 1 h at 37 °C. After incubation, all PMP membrane samples were gently washed with DPBS, to deplete non-adherent HL-60 cells, and transferred into new wells containing EGM-2. Pictures were taken at 5 random regions (ROI) across the PMP membrane samples, using a fluorescence microscope (AxioVert A1, Zeiss, Jena, Germany). Adhered, labelled HL-60 cells were counted using the ImageJ software (particle counter plug-in). The results are provided as mean HL-60 cell count per cm^2^ averaged from independent triplicates. 

### 2.11. Thrombocyte Adhesion Assay

For the thrombocyte adhesion assay, a previously published protocol [50,51] was adapted. Human thrombocyte concentrate was obtained from anonymized healthy donors undergoing plateletpheresis at the blood bank of the Hannover Medical School, with informed consent and according to the regulation of the local ethics committee. The thrombocyte concentrate was stored in blood collection tubes (Sarstedt, Nümbrecht, Germany) containing 1.6 mg ethylenediaminetetraacetic acid (EDTA) per mL blood. The thrombocyte suspension was diluted 1:10 with HEP-Buffer (140 mM NaCl, 2.7 mM KCl, 3.8 mM HEPES, 1 mM EGTA, pH 7.4, 15 ng/mL Iloprost), incubated for 10 min at RT and centrifuged at 800 × *g* for 20 min. The sedimented platelets were gently resuspended in 4% paraformaldehyde solution and fixed for 10 min. After 1:1 dilution with PBS fixed platelets were centrifuged at 800 × *g* for 10 min. The sedimented platelets were resuspended in 1:20 PBS/Sudan Black B (SBB, in 70% ethanol) and stained for 60 min at RT. Afterwards, platelets were centrifuged and washed three times to deplete the residual SBB. iPSC-EC samples, with or without deliberate TNFα activation, were incubated with 1.33 × 10^7^/cm^2^ SBB-stained thrombocytes added to the culture medium of the samples on a rocking platform for 30 min. PMP membrane samples, incubated with pooled normal blood plasma (Precision BioLogic Inc., Dartmouth, NS, Canada) for 6 h, were used in parallel as a positive control. Following incubation, all PMP membranes were removed from the well plate and drawn through PBS (300 mL) to remove unbound platelets, and placed into new wells containing 500 µL DMSO, with the platelet-exposed side facing the well bottom. After 30 min SBB-containing DMSO was transferred into wells of a 96 well plate (in duplicate) to measure the absorbance at 595 nm. Serial dilutions of DMSO containing defined numbers of SBB-stained platelets were measured in parallel, to generate a standard curve. Data is depicted as the mean thrombocyte count per cm^2^ with standard deviation (*n* = 3). 

### 2.12. Real-Time Reendothelialization Assay

Confluent endothelial cell monolayers were established on FN-coated PMP membranes mounted in the wells of 6-well plates. Using a 200 µL pipette tip, one scratch through the center of each monolayer was applied, thereby creating a cell-free area. Time-lapse microscopy phase contrast pictures from one scratched region per monolayer were taken in 7 min intervals for 12 h under standard culture conditions (5% CO_2_, humid atmosphere at 37 °C) using a microscope that was equipped with an incubation unit (AxioObserver Z1, Zeiss, Germany). The cell-free area of the scratched region was measured in pictures obtained directly at the start of the experiment and 12 h later using ImageJ (mask tool). The reendothelialized area was calculated by subtraction of the measured area after 12 h from the area measured at the start. Results are provided as mean reendothelialized area (µm^2^) per minute, deducted from 3 independent samples. 

### 2.13. Statistical Analysis

Statistical analysis was performed using GraphPad Prism Version 8 for Windows. Flow cytometry, qRT-PCR and leukocyte adhesion data were analyzed with the unpaired t-test. Reported values are given as means and standard error of mean, or standard deviation, where indicated. For the comparison of multiple groups compared in the scratch assay, one-way ANOVA with Tukey’s post hoc test was applied and values are given as means and standard deviation. Dunnett’s multiple comparison test was used to find differences between groups analyzed in the thrombocyte adhesion assay. Differences were considered significant at *p* < 0.05. 

## 3. Results 

### 3.1. Efficient Endothelialization of HFM

**Figure 1:**
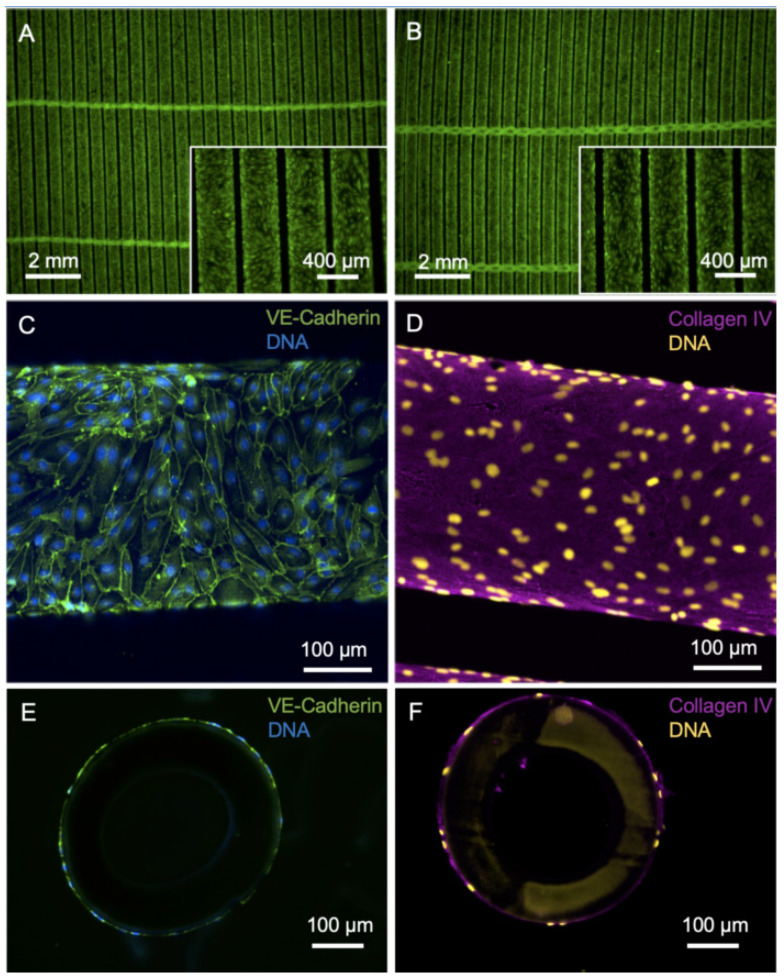
Imaging of HFM after endothelialization with iPSC-EC1. (**A**,**B**) Viable calcein-positive iPSC-ECs (green) seeded on fibronectin-coated HFM form a confluent monolayer around the individual fibers of the HFM. A: Top view, B: bottom view. (**C**) iPSC-ECs are interconnected via VE-cadherin (green). (**D**) Basal-lamina-like matrix envelops iPSC-EC-seeded hollow fibers, indicated by de novo synthesized collagen type-IV (magenta). (**E**,**F**) Nuclei counterstained with DAPI and presented in false colors (yellow).

With the primary goal of improving the longevity of gas exchange membranes in clinical use and with the vision of developing a BHL, we pursued the strategy to reduce the material’s thrombogenicity and the tendency to clogging by populating the surface of HFM with a confluent monolayer of iPSC-derived ECs (Figure 1). Forty-eight hours after EC seeding at a density of 2.04 × 10^5^ iPSC-ECs/cm^2^, the HFM showed a confluent colonization of viable calcein-positive ECs, both from the top (Figure 1A) and from the bottom (Figure 1B) view. iPSC-derived ECs from Donor1 showed a typical cobblestone morphology and a typical pattern of the major EC adhesion molecule VE-cadherin (CD144) with location at junctions between ECs indicating intact cell–cell contact integrity (Figure 1C). De novo formed collagen type-IV, which is a major constituent of the EC-specific basal lamina, was evenly dispersed at the abluminal surface of HFM (Figure 1D). Cross-sections of the HFM confirmed that ECs and collagen type-IV enveloped the fibers of the HFM (Figure 1E,F). 

### 3.2. iPSC-ECs on PMP Membrane Are in a Non-Activated State While Remaining Physiologic Responsive to TNFα Stimulation

**Figure 2:**
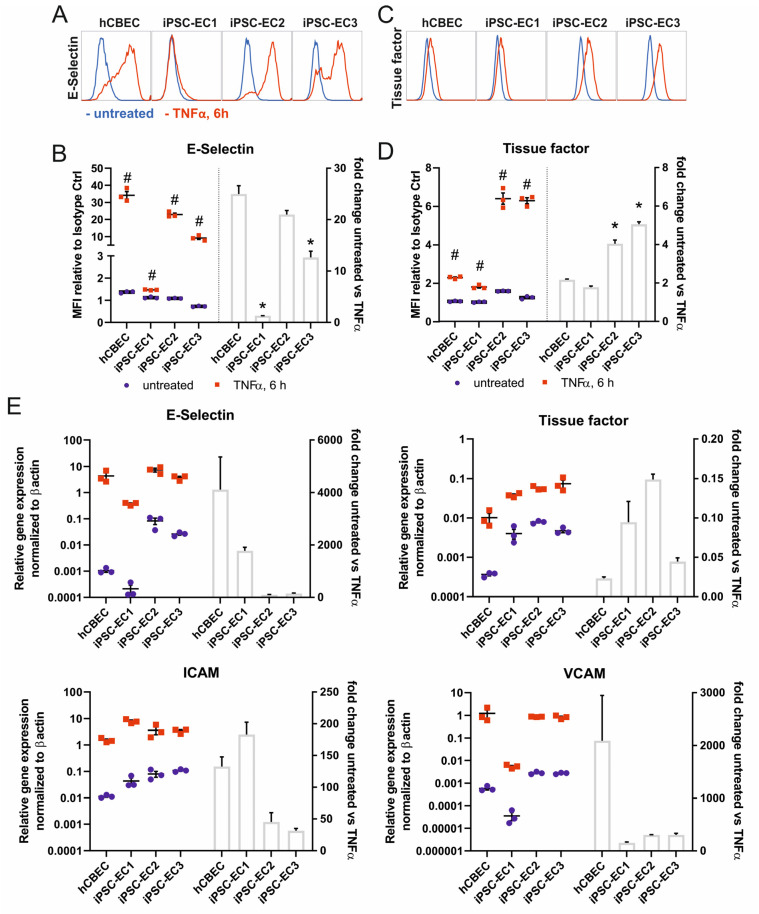
iPSC-derived ECs seeded on fibronectin-coated PMP membranes show upregulation of markers of inflammation and hemostasis upon stimulation with 10 ng/mL TNFα for 6 h. (**A**) Flow cytometry analysis of E-selectin (CD62E) surface expression. Representative histograms of untreated (blue histograms) vs. TNFα-treated (red histogram) ECs. (**B**) Quantification of mean fluorescence intensities (MFI, represents geometric mean normalized to corresponding isotype controls) of E-selectin expression with flow cytometry (left) and calculated fold-change of untreated vs. TNFα-treated ECs (right). (**C**) Representative histograms of flow cytometry analysis ECs for tissue factor (CD142) surface expression. (**D**) Quantification of mean fluorescence intensity (MFI) of tissue factor surface expression with flow cytometry (left) and calculated fold-change of untreated vs. TNFα-treated ECs (right). (**E**) qPCR quantification of activation-relevant marker genes E-selectin, tissue factor, ICAM-1 and VCAM-1. The mRNA expression was normalized to the expression of β-actin using the ΔCt-method. All data are representative of 3 technical replicates for each EC type. Reported values are given as means ± standard error of mean. Differences were considered significant at *p* < 0.05. # Significantly different TNFα-treated vs. untreated. * Fold change significantly different vs. hCBECs.

We investigated whether iPSC-ECs in cultures on the fibronectin-coated PMP membrane are present in their non-activated state, and whether they are able to show a physiologic pro-coagulative, pro-thrombogenic and pro-inflammatory response, comparable to the control cells. For these experiments we used flat fibronectin-coated PMP membranes made of the same material as the HFM, which are used in membrane oxygenators. The ECs were able to adhere to this surface and formed a confluent monolayer, which was stable for up to 14 days under standard culture conditions (Supplementary Figure S2). Monolayers, derived from three independent iPSC-lines were confluent after 48 h and were compared to hCBECs as a primary cell control in their level of E-selectin expression, indicating the pro-inflammatory response, and of tissue factor as a cell surface bound protein with pro-coagulative properties. Both factors were analyzed with and without stimulation with TNFα for 6 h using flow cytometry and realtime quantitative polymerase chain reaction. Flow cytometry analysis detected in all tested iPSC-EC lines and the hCBECs, a significant increase in the expression of E-selectin and tissue factor when iPSC-ECs and hCBECs were stimulated with TNFα. This correlated with the results obtained via realtime qRT-PCR, where a pronounced upregulation of the respective genes was also measured, when treated with TNFα (Figure 2A–E). This finding indicates the general functionality of the cells on the membrane in these parameters. Regarding the treatment of iPSC-EC1s, a lower E-selectin response was observed. We would not overinterpret differences in the level of upregulation, because only one timepoint (6 h of TNFα stimulation) was analyzed here, but different cell sources can have different time kinetics of the peak expression of the parameters examined, which was not considered here. The expected TNFα-controlled mRNA upregulation also applied for the endothelial cell adhesion molecules ICAM-1 and VCAM-1, which we assessed as a sign of a functional endothelial cell layer on the gas exchange membranes.

### 3.3. Leukocyte Adhesion Assay Confirms the Non-Inflammatory Status of iPSC-EC Monolayers 

**Figure 3:**
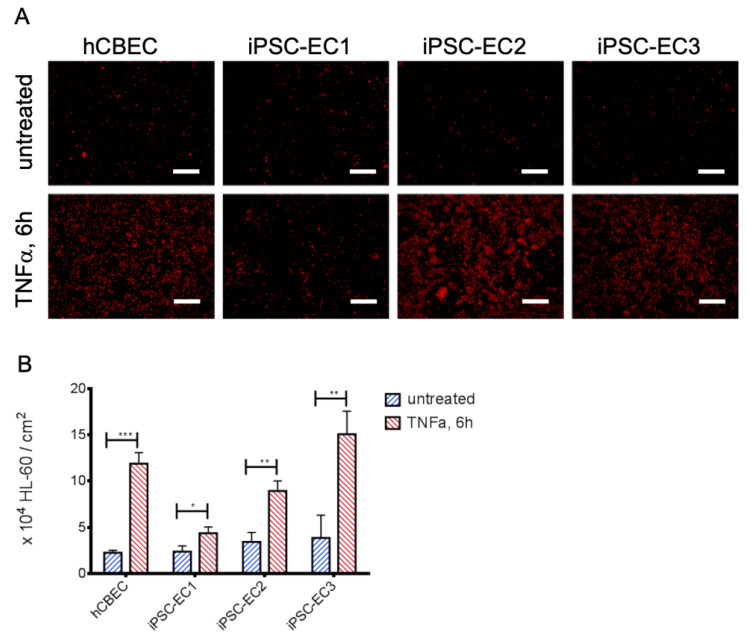
iPSC-ECs on PMP membranes show different levels of leukocyte adhesion after TNFα treatment. (**A**) Exemplary images of cell tracker red-stained HL-60 leukocytes (red) on iPSC-EC endothelialized gas exchange membranes under standard condition and after treatment with TNFα for 6 h. Except for monolayers established from iPSC-EC1s, appreciably increased numbers of HL-60 cells adhered to EC-monolayers incubated with TNFα. Scalebar: 250 µm. (**B**) Quantification of adhered HL-60 leukocytes. Reported values are given as means ± standard deviation. Significant differences between untreated and TNFα-stimulated groups were marked with * at *p* < 0.05, ** when *p* < 0.01 and with *** when *p* < 0.001.

Analysis of protein and gene expression indicated a tendency towards a lower abundance of E-selectin after TNFα-stimulation on iPSC-EC1s. Since E-selectin is a potent mediator for leukocyte adhesion, we sought to confirm this finding on a functional level. Therefore, we assessed the inflammatory status of confluent EC-monolayers on gas exchange membranes by using a leukocyte adhesion assay (Figure 3). When the leukemia cell line HL-60 was added to the membrane-seeded iPSC-ECs and hCBECs, a significantly higher number of HL-60 cells adhered after activation of the EC monolayers with TNFα. The most prominent response to TNFα with regard to leukocyte adhesion was obtained with monolayers from iPSC-EC3 monolayers (TNFα activated: 15.06 ± 2.50 HL-60/cm^2^ vs. untreated: 3.85 ± 2.47 HL-60/cm^2^, *p* < 0.01) followed by the hCBEC (TNFα activated: 11.89 ± 1.20 × 10^4^ HL-60/cm^2^ vs. untreated: 2.24 ± 0.28 × 10^4^ HL-60/cm^2^, *p* < 0.001). The difference in attached HL-60 cells between activated and non-activated iPSC-ECs was least pronounced for iPSC-EC1 monolayers (TNFα activated: 4.37 ± 0.66 × 10^4^ HL-60/cm^2^ vs. untreated: 2.38 ± 0.62 × 10^4^ HL-60/cm^2^, *p* < 0.05). These results were in line with those obtained by E-selectin protein- and gene expression analyses, as among the TNFα activated iPSC-ECs and hCBECs the number of adhered HL-60 cells was also lowest for iPSC-EC1.

### 3.4. iPSC-ECs Improve HFM Hemocompatibility

**Figure 4:**
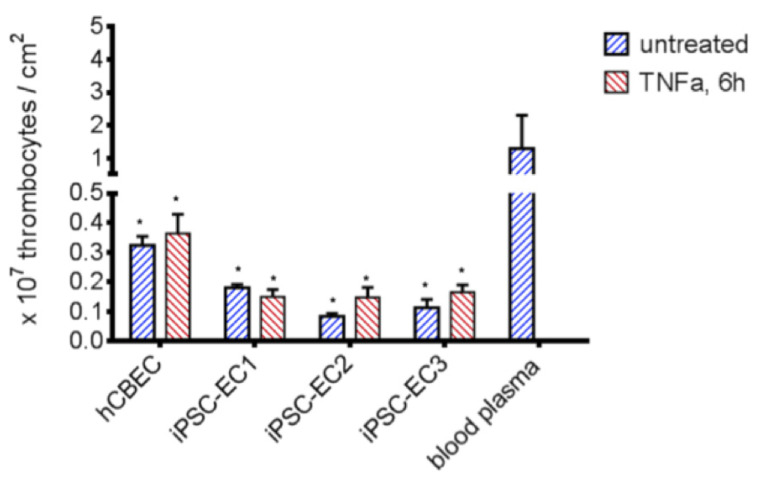
Reduced platelet adhesion to PMP membranes endothelialized with iPSC-ECs. Significantly less thrombocytes adhered to endothelialized samples, compared to blood plasma coated PMP membranes (*p* < 0.05). A higher platelet count could be detected when iPSC-EC2, iPSC-EC3 and hCBEC were activated with TNFα for 6 h, while differences were statistically not significant. All data represent the means of independent triplicates for each EC type and treatment. Dunnet’s multiple comparison test, differences to the blood plasma group were considered significant at *p* < 0.05 (*).

The suppression of platelet adhesion to materials is a critical factor in the development of a BHL to prevent thrombus formation and its lethal consequences. We tested the potential of plain versus endothelialized PMP membranes to prevent thrombocyte adhesion in the absence and presence of pro-thrombotic TNFα (Figure 4). In order to simulate realistic conditions of membrane clogging after a 6-h ECMO run, we soaked the plain membranes in blood plasma which can be assumed to contain pro-thrombogenic factors. 

Compared to the blood plasma-immersed PMP membrane (1.29 ± 1.02 × 10^7^/cm^2^ adhered thrombocytes), significantly lower numbers of adhered thrombocytes were found on PMP membranes, when an endothelial cell monolayer was present. A higher number of adhered platelets was counted following the TNFα stimulation of the ECs iPSC-EC2, iPSC-EC3 and hCBECs, but not iPSC-EC1. However, the differences in adhered thrombocytes between all ECs under standard conditions and after TNFα stimulation were statistically not significant. The lowest number of thrombocytes was found on monolayers established from iPSC-EC2 under standard conditions (0.08 ± 0.01 × 10^7^/cm^2^), while in general platelet counts on iPSC-EC monolayers were lower than on hCBEC monolayers. This data provided evidence that the endothelialization strategy was effective in reducing thrombocyte adhesion to the gas exchange membrane, and that iPSC-ECs had a tendency to be superior to hCBECs regarding this feature. 

### 3.5. Regenerative Capacity of PMP Membrane Seeded iPSC-ECs to Reendothelialize Scratched Monolayer Areas

Current approaches to reduce the thrombogenicity of blood-contacting surfaces, established by various manufactures of ECMO oxygenators, are found in coating the surfaces with anti-coagulative or anti-adhesive molecules, which are prone to wearing or can become masked over time by blood proteins.

**Figure 5:**
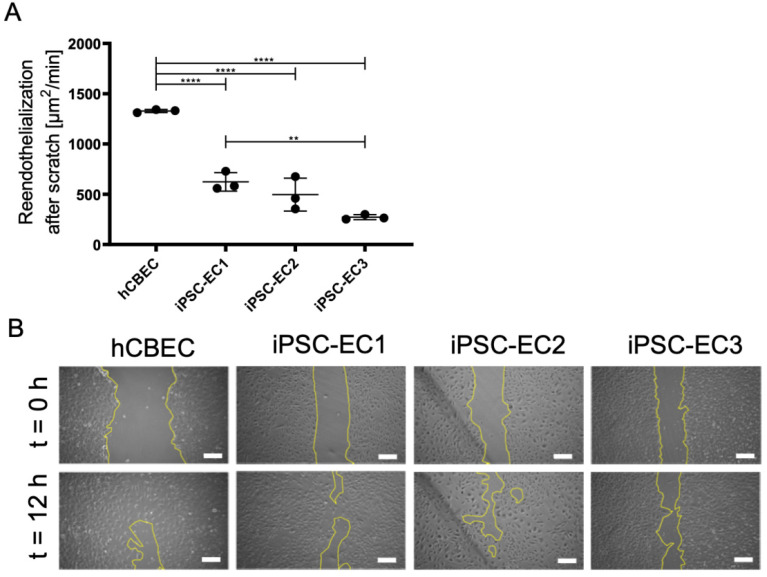
Regenerative capacity of endothelial monolayers on PMP gas exchange membranes. (**A**) Recovered area per minute. Results are given as means from independent triplicates with standard deviation. Analysis for statistically significant differences was performed using one-way ANOVA with Tukey’s post hoc test. Differences were considered significant at *p* < 0.01 (**) and *p* < 0.0001 (****). (**B**) Representative phase contrast pictures showing the scratched area at start (t = 0) of the experiment and after 12 h. Yellow lines indicate the cell-free area. Scale: 250 µm.

A major advantage of the EC-monolayer over the inanimate coatings lies in the regenerative capacity of the cells, either by migration or cell division. In order to demonstrate this important behavior and to identify potential differences in the time needed for the different iPSC-ECs and hCBECs to repopulate the PMP membrane, a monolayer scratch-assay was carried out (Figure 5). 

Wound closure time of scratched areas was significantly faster in monolayers established from hCBECs (1328.0 ± 15.5 µm^2^/min), than in monolayers established from iPSC-ECs (*p* < 0.01). Among the iPSC-ECs, fastest repopulation was observed with iPSC-EC1 cells (622.9 ± 92.1 µm^2^/min), while the lowest recovery rate was measured in monolayers established from iPSC-EC3 (272.3 ± 24.6 µm^2^/min). For the iPSC-EC2 monolayer a recovery rate of 426.1 ± 163.7 µm^2^/min was determined. However, the proven regenerative capacity of the anti-thrombogenic endothelial monolayer suggests that the whole endothelialized BHL oxygenator could mitigate thrombus-caused device clogging significantly, thereby justifying long-term application and even device implantation.

## 4. Discussion

Due to the increasing discrepancy between potential organ donors to organ recipients and the missing alternative therapy option to LTx, we aim for the development of the BHL as alternative to LTx, but also as final destination therapy. Indeed, BHL development is based on the well-established ECMO technology, but for reliable and long-lasting application, complete hemocompatibility of all artificial materials needs to be achieved. For this, a very large number of ECs need to be generated, because besides the housing and tubing, the whole oxygenator with the integrated HFM comprises a blood contacting area of more than 2.5 m^2^, which needs to be endothelialized. 

Here we provide the proof-of-principle that iPSC-derived ECs can be used for efficient endothelialization of PMP HFM to shield the artificial surfaces from the patients’ blood. Similar results have already been published using primary EC sources [20,21,23], such as HLA-silenced “immuno-invisible” cells, but the advantage of iPSC-ECs over these cells, however, is that they can be produced in virtually infinite quantities in stirred tank bioreactors [40,52] and represent a potentially autologous cell source with complete histocompatibility. We generated iPSC-ECs from three different wildtype iPSC lines using our scalable EC-differentiation protocol [40] and selected one of these sources to firstly show that the iPSC-ECs are capable of forming viable and confluent monolayers on fibronectin-coated PMP HFMs. In this case, fibronectin was applied, as it provides a near-physiologic matrix and contains specific peptide sequences, e.g., RGD sequences, that are recognized by integrins [53] expressed on the endothelial cell surface. In addition, EC adhesion to fibronectin coating on gas exchange membranes has been reported to be efficient in the past by other groups, as well [25]. Viable ECs grew over the entire area around the fibers and formed characteristic VE-cadherin cell-cell contacts and a collagen IV-containing EC-like basement membrane. Cryosections of these samples confirmed the confluence of the monolayer around the whole fiber circumference, while the fiber was also wrapped completely with collagen type-IV. This observation holds promise for the flow-resistant long-term adherence of cells to the fiber surface. Thus, the fibronectin pre-coating might be relevant for the initial cell seeding only, which will be amended over time by a de novo-built basal lamina-like matrix, representing another hallmark of the EC monolayer as a regenerative and self-sustaining surface coating. A stable and self-organizing coating may also be crucial for the resistance of the EC monolayer to the flow conditions existing in future BHL. However, earlier studies assessing hCBEC monolayer on differently precoated HFM indicated that the EC monolayer is in principle capable of withstanding shear stress that is generated during the flow conditions within the BHL [20], which is why we expect the same properties for iPSC-ECs. 

It is prerequisite for the reliable long-term application of the implantable BHL that the seeded ECs preserve the endothelial-specific, physiologic non-thrombogenic phenotype to shield off thrombocytes and avoid the activation of the coagulation cascade, which will result in severe thrombus formation, resulting in the life-threatening exchange of the BHL. Additionally, the endothelial-specific, physiologic non-inflammatory status should also be preserved, as inflammatory responses will result in the activation of the complement system and cytokine activation with the release of inflammatory mediators, leading to sepsis. Furthermore, it can also lead to both, the increased permeability of the EC monolayer on the HFM, which may reduce their flow-stability, and also to the induction of the cell death [54]—both resulting in the uncovering of the HFM and presenting the potentially pro-thrombogenic, artificial HFM surface to the circulating blood. 

Therefore, we tested the iPSC-EC monolayers on the PMP membrane for their expression of markers indicating an activation towards the pro-inflammatory and pro-thrombotic phenotype in presence or absence of TNFα. As expected, all iPSC-ECs showed a significant physiologic upregulation in the expression of E-selectin and tissue factor when deliberately challenged with TNFα, however, the different cell lines displayed a different degree of responsiveness. This result was not unexpected at this point, because there may be cell line differences with regard to the TNFα response, but time kinetics of factor upregulation was not carried out here. Therefore, it could also be that the peak expression was not reached or had already been exceeded after 6 h of TNFα stimulation. It must be considered that different clones can differ in terms of their proliferation, proinflammatory response and function. Nevertheless, reduced or delayed activation responses of the iPSC-ECs may be even advantageous, as the monolayer could tolerate higher cytokine levels presented in the patient’s blood [11]. This can be tested in vitro prior to their clinical application. In summary, it can be stated from this experiment that the ECs on the PMP membrane retain a physiologic non-activated phenotype and show physiological upregulation of characteristic markers upon treatment with inflammatory stimuli. These findings are also in line with the results obtained from the functional leukocyte adhesion assay. 

In addition, the iPSC-EC monolayers established on the gas exchange membrane were also capable of reducing the number of surface-adhering thrombocytes, which is one of the most crucial and desired properties that ECs need to possess for identifying them as suitable in the development of the implantable BHL [19]. As thrombocyte adhesion and subsequent thrombotic occlusions are most frequently observed complications in contemporary oxygenators [8,55], masking the blood-contacting surfaces of the implantable BHL oxygenator with an iPSC-EC monolayer may reduce, or even abdicate the necessity for a highly dosed and meticulously controlled systemic anti-coagulation therapy of future patients. 

While the iPSC-ECs analyzed in this study were tested positive for this anti-thrombogenic attribute, additional genetic engineering techniques may be used in the near future to either foster their anti-thrombogenic behavior, or render them less sensitive towards pro-coagulative stimuli. Those techniques are already available, e.g., to reduce the immune rejection response to allogeneic cells by silencing HLA-Class I [56]. In future studies, this technology may be transferrable to iPSC-ECs, to further increase their expression of anti-thrombogenic proteins like thrombomodulin [46,47] or nitric oxide synthase 3 (eNOS) [44,45].

A further prominent advantage of a viable EC monolayer is the property to regenerate temporary unseeded, or rather “wounded” areas, which can appear within BHL application due to necessary cell regeneration or flow-dependent cell loss. While pure molecule-based coatings are prone to wearing-off and degradation, ECs are capable of detecting the absence of a neighboring cell and amend the gaps in the monolayer by directed migration and proliferation. In order to assess this property, monolayers from different iPSC-ECs and hCBECs on PMP membranes were subjected to a scratch assay. All tested EC monolayers were able to repopulate the scratched area, but with deviations in the time required for wound closure. Overall, however, this was a promising result with regard to the desired regenerative potential of a BHL device in long-term use.

In summary, we have provided additional evidence that lung support systems could utilize a functional, self-regenerating biohybrid approach. In this context, iPSC-derived ECs could be the cell source of choice for membrane endothelialization, since they are currently the most flexible cell source, with several advantages over autologous primary ECs, but also allogeneic ECs. Most importantly, it should be feasible to gain the high numbers of clinically relevant ECs needed to endothelialize the large surface areas of the implantable BHL. The promising results of this study justifies further studies on assessing the iPSC-ECs under real-scenario workload conditions (e.g. gas transfer and blood flow) and during BHL long-term application.

## Supplementary Materials

The following are available online at https://www.mdpi.com/article/10.3390/mi12080981/s1, Figure S1: HFM seeding procedure, Figure S2: Long-term cultivation of hCBECs and iPSC-ECs, Table S1: List of primary and secondary antibodies, Table S2. List of primer pairs for qRT-PCR.
