# Supplementary material for: Towards Biohybrid Lung: Induced Pluripotent Stem Cell Derived Endothelial Cells as Clinically Relevant Cell Source for Biologization
Source: Micromachines (Basel). 2021 Aug 19;12(8):981. doi: 10.3390/mi12080981 (PMC8401467; doi:10.3390/mi12080981)
Supplement: Supplementary file 1 [file micromachines-12-00981-s001.zip › micromachines-1281827-supplementary.pdf]

micromachines

MDPI

Supplementary Materials:

Towards Biohybrid Lung: Induced Pluripotent Stem Cell derived Endothelial Cells as Clinically Relevant Cell Source for Biologization

Table S1. List of primary and secondary antibodies.

	Dilution	Vendor	Application
APC-labelled anti-E-selectin (CD62E)-IgG	1:5	BD Biosciences	FACS
PE-labelled anti-tissue factor (CD142)-IgG	1:5	BD Biosciences	FACS
APC-labelled isotype CTRL	1:5	BD Biosciences	FACS
PE-labelled isotype CTRL	1:5	BD Biosciences	FACS
anti-hVE-cadherin	1:50	AbD Serotec	IF
anti-collagen-IV (clone CIV 22)	1:50	Dako	IF
goat anti-mouse Cy2	1:125	Jackson ImmunoResearch	IF
donkey anti-rabbit Cy3	1:125	Jackson ImmunoResearch	IF

Supplementary Materials and Methods:

RNA isolation and quantitative real-time polymerase chain reaction

Total RNA was prepared from cells using Trizol (Thermo Scientific). Contaminating DNA was digested by DNAse I (Stratagene) for 15 min at 37 °C followed by phenol/chloroform extraction. After ethanol precipitation, 200 ng RNA was used for random-primed cDNA synthesis with SuperScript II Reverse Transcriptase (Thermo Scientific). Furthermore, 1 µL of 1:5 diluted cDNA was amplified with the Absolute qPCR SYBR Green Mix (Thermo Fisher #AB1159A) and 1 mM of each primer in a 25 µL reaction using a Mastercycler ep realplex2 (Eppendorf). PCR conditions included an initial denaturation step at 95 °C for 15 min, followed by 40 cycles of denaturation at 95 °C for 1 min, annealing at TA for 1 min, and polymerization at 72 °C for 1 min. Size of amplicons and absence of non-specific products were controlled by melting curves. Relative expression levels compared to the reference gene β-actin were calculated using the ΔCt-method. All PCR reactions were performed in duplicate.

Table S2. List of primer pairs for qRT-PCR.

Gene name	Gene Bank ID	5'-Primer	Product size (bp)	Annealing Temp, TA	Reference
**E-Selectin**	gi:182047	ATCCAGCCAATGGGTTCGTG<br>GAAGGCTCTGGGCTCCCATT	114	61	Hess et al. 2010
**ICAM-1**	gi:167466197	CTACCTCTGTCGGGCCAGGA<br>AGGCCTGCAGTGCCCATTAT	132	61	Hess et al. 2010
**VCAM-1**	gi:18201907	GGCGCCTATTACCATCCGAAA<br>GAGCACGAGAAGCTCAGGA-<br>GAA	156	61	Hess et al. 2010
**Tissue factor**	gi:10518499	CCCGAACAGTTAACCGGAAGA<br>GGAGTTCTCCTTCCAGCTCTGC	191	61	Hess et al. 2010
**Thrombomodulin**	gi:40288292	GCCCATGGGAGCTGGTTAGA<br>GGCCTGACTTGGCCTGCTAC	190	61	Hess et al. 2010
**ß-Actin**	gi:168480144	ATGTTTGAGACCTTCAACAC<br>CACGTCACACTTCATGATGG	176	61	Hess et al. 2010

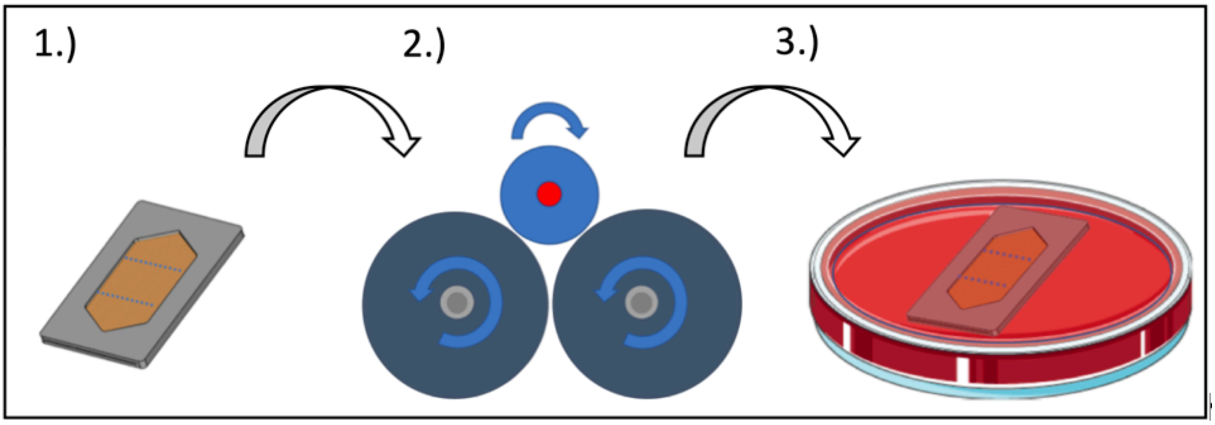

Figure S1. HFM seeding procedure. 1.) Samples were cut out from a larger hollow fiber membrane mesh, immersed in FN solution overnight and mounted in polycarbonate frames. 2.) Framed samples were then placed in 50 mL syringes that were filled with 25 mL of the iPSC-EC suspension and rotated with 1 rpm on a roller device for 4 h, at 37 °C. 3.) After rotation, samples were transferred into culture dishes containing culture medium, for cultivation under standard conditions.

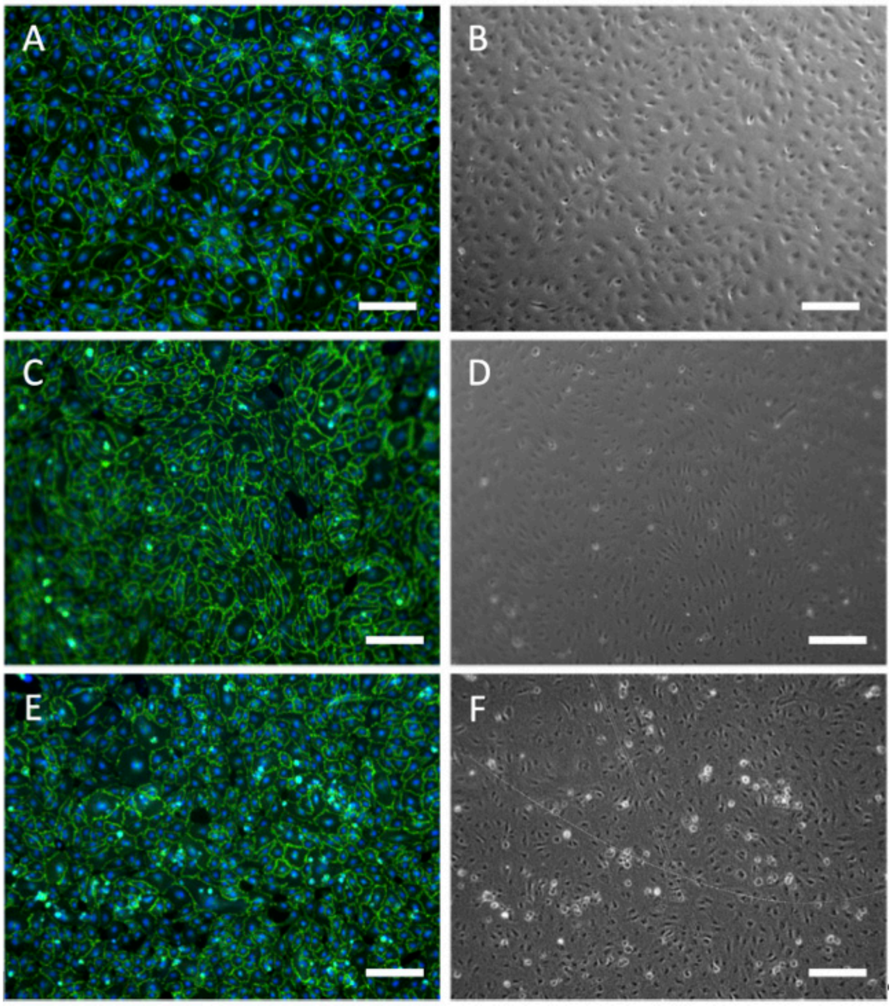

Figure S2. Long-term cultivation of hCBECs and iPSC-ECs. After seeding on FN-coated PMP-films, hCBECs (**A**,**B**) and iPSC-EC 2 (**C**,**D**) and iPSC-EC 3 (**E**,**F**) were cultured under standard static culture conditions for 14 days. After fixation with 4% PFA, monolayers were forwarded to immunofluorescence detection of VE-cadherin (**A**,**C**,**E**) (green) and nuclei (blue). Phase contrast pictures were acquired in parallel (**B**,**D**,**F**). Scale bar: 250 μm.
